# Membrane regulation of 15LOX-1/PEBP1 complex prompts the generation of ferroptotic signals, oxygenated PEs

**DOI:** 10.1016/j.freeradbiomed.2023.09.001

**Published:** 2023-09-09

**Authors:** Thiliban Manivarma, Aleksandr A. Kapralov, Svetlana N. Samovich, Yulia Y. Tyurina, Vladimir A. Tyurin, Andrew P. VanDemark, Wieslaw Nowak, Hülya Bayır, Ivet Bahar, Valerian E. Kagan, Karolina Mikulska-Ruminska

**Affiliations:** a Institute of Physics, Faculty of Physics, Astronomy and Informatics, Nicolaus Copernicus University in Torun, Torun, Poland; b Department of Environmental and Occupational Health and Center for Free Radical and Antioxidant Health University of Pittsburgh, Pittsburgh, PA, USA; c Department of Biological Sciences, University of Pittsburgh, Pittsburgh, PA, USA; d Department of Pediatrics, Division of Critical Care and Hospital Medicine, Redox Health Center, Vagelos College of Physicians and Surgeons, Columbia University Irving Medical Center, New York, NY, USA; e Laufer Center for Physical and Quantitative Biology and Department of Biochemistry and Cell Biology, Stony Brook University, New York, USA; f Department of Radiation Oncology, University of Pittsburgh, Pittsburgh, PA, USA; g Department of Chemistry, University of Pittsburgh, Pittsburgh, PA, USA; h Department of Pharmacology and Chemical Biology, University of Pittsburgh, Pittsburgh, PA, USA

**Keywords:** Ferroptosis, 15-Lipoxygenase, PEBP1, Lipid peroxidation, Membrane effect

## Abstract

Ferroptosis is a regulated form of cell death, the mechanism of which is still to be understood. 15-lipoxygenase (15LOX) complex with phosphatidylethanolamine (PE)-binding protein 1 (PEBP1) catalyzes the generation of pro-ferroptotic cell death signals, hydroperoxy-polyunsaturated PE. We focused on gaining new insights into the molecular basis of these pro-ferroptotic interactions using computational modeling and liquid chromatography-mass spectrometry experiments. Simulations of 15LOX-1/PEBP1 complex dynamics and interactions with lipids revealed that association with the membrane triggers a conformational change in the complex. This conformational change facilitates the access of stearoyl/arachidonoyl-PE (SAPE) substrates to the catalytic site. Furthermore, the binding of SAPE promotes tight interactions within the complex and induces further conformational changes that facilitate the oxidation reaction. The reaction yields two hydroperoxides as products, 15-HpETE-PE and 12-HpETE-PE, at a ratio of 5:1. A significant effect of PEBP1 is observed only on the predominant product. Moreover, combined experiments and simulations consistently demonstrate the significance of PEBP1 P112E mutation in generating ferroptotic cell death signals.

## 1. Introduction

Ferroptosis is an iron-dependent programmed cell death that occurs upon massive accumulation of lipid hydroperoxides [1] – a unique feature that distinguishes it from other cell death programs [2]. It is one of the most preserved and ancient forms of cell death characteristic of all domains of life, including evolutionarily remote species [3–5]. It is implicated in a broad range of diseases [3,6], including neurodegenerative diseases (e.g., Alzheimer’s [7,8] and Parkinson’s [9,10] where iron-dependent accumulation of lipid peroxides is enhanced), cerebello-cortical atrophy, sepsis, or bacterial and viral diseases [11,12], as well as multiple organ inflammation in COVID-19 [13–16], kidney failure, brain trauma, and asthma [17–19]. Targeted induction of ferroptosis has been proposed to have a potential in cancer therapy and in treating auto-immune diseases [20–22].

Three major factors trigger ferroptosis in cells: accumulation of redox-active iron, lipid peroxidation, and thiol dysregulation. Lipid peroxidation occurs through enzymatic or non-enzymatic mechanisms [23,24] and depends on reactive oxygen species, iron, and polyunsaturated phospholipids (PLs) rich in bis-allylic carbons. Here we focus on the generation of pro-ferroptotic lipid peroxides catalyzed by a complex formed between 15-lipoxygenase (15LOX) (a non-heme Fe-enzyme) and phosphatidylethanolamine-binding protein 1 (PEBP1). In 15LOX, a Fe^2+^ to Fe^3+^ oxidation generates a ferric iron-(Fe^3+^)-hydroxy species that triggers a stereoselective hydrogen abstraction from the polyunsaturated fatty acids (PUFAs) substrate, which is thus oxidized to a carbon-centered radical capable of reaction with unactivated molecular oxygen to yield a peroxyl radical as the primary oxygenated intermediate [25,26]. Iron is also essential for the proper positioning of the substrate enabling the oxygenation reaction to take place. While different types of PUFAs are involved in peroxidation, two fatty acyls – arachidonoyl (C20:4, AA) and adrenoyl (C22:4, AdA), esterified into PE are major substrates of the 15LOX/PEBP1 complex [19,27,28].

There are two isoforms of 15LOX, 15LOX-1 and 15LOX-2. These isoforms exhibit distinct expression in different tissues and generate partially diverse oxygenation products [25,29]. 15LOX-1 and -2 share high structural similarity with 36% sequence identity [4,5]. Either isoform binds to PEBP1 complexed with 15LOX, which enables PL peroxidation leading to ferroptotic cell death [19]. PEBP1 binding changes the substrate specificity of 15LOX to endow catalytic competence towards the esterified PUFA-PL, stearoyl/arachidonoyl-PE (SAPE), and generate hydroperoxy-PEs [19,30–33]. However, the atomic-level mechanism of PL peroxidation by 15LOX/PEBP1 remains to be elucidated.

The role of the PL membrane had been initially underestimated and considered only as a mere scaffold for facilitating the binding of membrane proteins. Numerous studies in the past years have shown that the lipids themselves regulate the localization and activity of many membrane-associated/peripheral proteins [34]. Among such proteins exhibiting membrane-mediated activities, we distinguish cytochromes [35–37] or phospholipases [10,38], which possess an ability to selectively associate with the PL bilayer, facilitating the extraction of PL substrates from the membrane to initiate the enzymatic reaction.

In the present study, we report the first computational study of the dynamics and interactions within the 15LOX-1/PEBP1 complex in the presence of a PL membrane that reveals the mechanism of SAPE substrate acquisition to the catalytic site of 15LOX, triggered by protein-membrane interactions. We confirmed the mechanistic model deduced from molecular dynamics (MD) simulations by mutagenesis studies using liquid chromatography-mass spectrometry (LC-MS) experiments, including the significance of PEBP1 P112E mutation in generating ferroptotic cell death signals. Moreover, we demonstrated the regulatory role of PEBP1 in the peroxidation of SAPE to the most predominant product, 15-HpETE-PE.

## 2. Methods

### Molecular docking.

In order to determine the structural model of the human 15LOX-1/PEBP1 complex, we applied a series of molecular docking simulations using the HDOCK server [39]. The initial structure of the human 15LOX-1 (Uniport ID: P16050) was obtained from the homology model of rabbit form (PDB ID: 2p0m [40]) using the Swiss-Model server [41]. The sequence identity between these sequences is >81%. To have an ensemble of PEBP1 structures, we used four monomers in our protein-protein docking approach (PDB ID: 1behA [42], 1behB, 1bd9A [42], 1bd9B). In total, we generated 100 hypothetical models for the 15LOX-1/PEBP1 complex which we narrowed down to four based on experimental findings [19], i.e. the effect mutations at P112 in PEBP1 and role of C-terminal helix of PEBP1 in the complex formation. These four computational models (called *Models 1*–*4*) were subjected to further investigation (see Supplementary Fig. S1A). Docking simulations were performed using the SMINA package [43] to identify the initial position of SAPE at the catalytic site of 15LOX-1 complexed with PEBP1, to be adopted in MD simulations of *Models 1* and *3*. We performed 5 runs for each system and selected SAPE conformations which had the highest binding affinity and placing the sn-2 chain C13 carbon atom of SAPE in a close proximity to the iron at the catalytic site as required for the peroxidation reaction.

### Molecular Dynamics simulations.

Full-atomic MD simulations were performed for *Models 1*–*4* using NAMD [44] package and the CHARMM [45] force field, and 2 fs time steps. The proteins were solvated with explicit water (TIP3P) at physiological salt concentrations. CHARMM force field parameters for bonded iron were obtained using Gaussian [46] (DFT B3LYP/6–31(d,p) method).

We performed two sets of simulations for each system, with and without membrane. Prior to productive runs without membrane, the following protocol was adopted: 0.2 ns of water equilibration, 10,000 steps of minimization, 0.35 ns of heating from 0 to 300 K, and 0.15 ns equilibration of the whole system. A cutoff of 12 Å for non-bonded interactions was applied. Langevin dynamics and the Langevin piston algorithm were used to maintain the temperature at 300 K and the pressure at 1 atm. For each system we performed >600 ns (3 MD runs, each >200 ns). We further eliminated *Model 4* which was highly unstable.

Simulations with the membrane composed of 1,2-dioleoy-sn-glycero-3-phosphocholine (DOPC, 50%), 1,2-dioleoy-sn-glycero-3-phosphoethanolamine (DOPE, 30%), and 1-stearoyl-2-arachidonyl-phosphoethanolamine (SAPE, 20%) were prepared in CHARMM GUI server [47]. We used the PPM/OPM [48] server to predict the orientation of the protein complex in the membrane. For each system, we performed 250 ns simulations (Supplementary Table 2). *Model 2* due to the increase in the distance between P112 and 15LOX-1 structure was eliminated from further consideration.

Next, for *Models 1* and *3*, we performed 200 ns long simulations of the 15LOX-1/PEBP1 complex with SAPE as a substrate bound to the catalytic site. For *Model 1,* we further performed 75 ns simulations with point mutations in PEBP1 (P112E, H86A, H86E, and P74L). We used our own scripts in ProDy [49] API and VMD [50] for analyses and visualization. The Adaptive Poisson–Boltzmann Solver (ABPS) [51] software was used to predict the electrostatics.

#### PEBP1 expression and purification:

Full-length human PEBP1 and related mutants were cloned into a pET21-derived bacterial expression plasmid (EMD Millipore, Billerica, MA) modified to express PEBP1 with N-terminal His 10-and mRuby 2 tags [52]. All PEBP1 constructs were cloned into the modified pET21-mRuby2 vector by Gibson Assembly (New England Biolabs, Billerica, MA) using primers with homology at the upstream (sense) NdeI site (5′-GGTCTGAGGGGATACACTCA TATG-3′) and downstream (antisense) EcoRI site (5′-GCTTGTCGA CGGAGCTCGAATTC-3′) of the vector. Preparation of mutations, evaluation of clones, expression and purification of proteins were performed as described in [19]. Before performing experiments, PEBP1 was desalted into 5 mM Bis-Tris (pH 6.5), 25 mM NaCl as described previously.

#### 15LOX-1 expression and purification:

Plasmids for expressing the catalytic domain (residues 112–663) of porcine 15LO1 was generously provided to us by Max Funk. Purification was carried out as previously described [19,53].

#### Cross-linking:

Recombinant human PEBP1 (2 μM) and porcine 15 LOX1 (2 μM) crosslinking was performed by incubation with 0.1% glutaraldehyde in 20 mM HEPES (pH 7.4) for 15 min. The reaction was stopped by addition of tris-HCl (pH 7.5) to final concentration 200 mM followed by ncubation for 15 min at room temperature. SDS PAGE of samples was performed in 7.5% running gel and proteins were stained by GelCode SilverSNAP kit (ThermoFisher Scientific). In some cases, proteins were electro-transferred to nitrocellulose membrane, blocked by 5% skim milk and PEBP was immunodetected using PEBP-specific antibodies (Santa Cruz, # SC-28837, 1:1000), HRP-conjugated goat anti-rabbit IgG H&L (Sigma, #A0545, 1:1000) as secondary antibodies and SuperSignal West Pico Substrate (ThermoFisher Scientific). The densities of protein bands were assessed by an open source image processing program: Image J.

#### Far-western blotting:

The interaction between PEBP and 15LOX *in vitro* was examined with far-western blotting. To assess the interaction of wild type and mutated PEBP1 with 15LO1, wt PEBP1 as well as mutants PEBP1 (4 μM) were separated on 10% Tris-glycine SDS-PAGE and electrically transferred to a nitrocellulose membrane. Proteins were renatured by incubation of the membrane in buffer (100 mM NaCl, 20 mM Tris (pH 7.6), 0.5 mM EDTA, 10% glycerol, 0.1% Tween-20, 2% skim milk powder and 1 mM DTT) containing decreasing concentration of guanidine HCl (6 M, 3 M, 1 M, 0.1 M, 0 M) and the membrane was blocked with 5% skim milk.

Then the membrane was incubated in 0.5 ml of protein-binding buffer (100 mM NaCl, 20 mM Tris (pH 7.6), 0.5 mM EDTA, 10% glycerol, 0.1% Tween-20, 2% skim milk powder and 1 mM DTT) containing 3 μM 15LOX-1 and DOPE/DOPC liposomes (1:1) (ratio DOPE to 15 LO1 25:1) at 4 °C overnight. Bound with membrane 15LOX-1 was immunodetected with 15LOX-specific antibodies (Life Span Biosciences Inc, #LS-111783, 1:2000) after 1.5 h incubation at room temperature. HRP-conjugated goat anti-rabbit IgG H&L (Sigma, #A0545,1:1000), was used as the secondary antibody, and bands were detected using SuperSignal West Femto Maximum Sensitivity Substrate (ThermoFisher Scientific)

#### Liposome preparation:

Liposomes of DOPC/SAPE (1:1) were prepared using Avanti^®^ Mini-Extruder. Briefly, 1,2-dioleoyl-PC (DOPC) and 1-stearoyl-2-arachidonyl-PE (SAPE) (Avanti Polar Lipids Inc.), lipids were dried with a stream of nitrogen gas and resuspended in 25 mM HEPES buffer, containing 100 μM diethylenetriaminepentaacetic acid (DTPA) pH 7.4 to achieve a final lipid concentration of 200 μM. The suspension was shaken vigorously and extruded through a polycarbonate membrane with 100 nm pores.

#### Lipoxygenase activity

was assessed by the formation of primary products of SAPE oxidation, 15-HpETE and 12-HpETE. Briefly, DOPC/SAPE liposomes were incubated with human recombinant 15LOX1 (0.04 μM) in the presence or in the absence of PEBP1 or PEBP1 mutant P112E (0.04 μM) for 2.5 min at 37 °C. To prevent the conversion of lipid hydroperoxides to secondary products during incubation, the HEPES buffer was saturated with oxygen. At the end of incubation lipids were extracted using Folch procedure [54] and analyzed by LC-ESI-MS/MS.

#### LC-ESI-MS/MS analysis of phospholipids:

LC-ESI-MS/MS analysis was performed on a Thermo Ultimate 3000 HPLC system coupled to a hybrid quadrupole-orbitrap mass spectrometer, (Q-Exactive, ThermoFisher Scientific) with an Xcalibur operating system. The instrument was operated in negative ion mode at a voltage differential of −4.0 kV and source temperature of 320 °C. Sheath gas and s-lens were set at 20 and 65, respectively. The resolution was set at 140,000 with a scan range of *m*/*z* 150–1800 and a user-defined mass tolerance of 5 ppm m/z values for the oxidized species are presented to 4 decimal places. MS/MS was performed in data dependent mode with HCD fixed at 24 and a resolution of 17,500. Non-oxidized and oxidized lipids were separated on a reverse phase Accucore C30 column (2.6 μm, 250 × 2.1 mm (ThermoFisher Scientific)) at a flow rate of 0.1 mL/min. Column temperature was set at 35 °C. The column was eluted using a gradient solvent system consisting of mobile phase A (acetonitrile/water, 50/50 v/v) and mobile phase B (2-propanol/acetonitrile/water, 85/10/5 v/v). Both mobile phases contained 5 mM ammonium formate and 0.1% formic acid. The gradient was performed as follows: 30%–70% B, 0–20 min; 70%–100% B, 20–55 min; 100% B, 55–70; 100%–30% B, 70–85 min; 30% B, 85–95 min for equilibration of the column. All gradients were linear.

## 3. Results

Docking and molecular dynamics simulations indicate that 15LOX-1/PEBP1 complex preferentially selects closed conformers in solution.

To establish a structural model for the human 15LOX-1/PEBP1 complex, we performed extensive molecular docking simulations and generated over 100 conformations. We used as input 15LOX-1 [40] and several crystallographic PEBP1 [42] structures (with pairwise root-mean-square deviations (RMSDs) of ~0.3 Å). Simulations revealed several hot spots on PEBP1, including A30, K47-R49, K80-Y81, D96-V102, D105, P112-K113, I137-H145, and the C-terminal helix D175-Y186 (Supplementary Fig. S1A). 15LOX-1 exhibited a preference for interfacial interactions at the F174-N192 (α2) and F583-L596 helices (Supplementary Table 1).

Based on our previous experiments [19] suggesting that PEBP1 residue P112 and C-terminal helix might be essential to association with 15LOX-1, we selected four models, *Models 1*–*4* (Supplementary Fig. S1B): (i) *Model 1*, proposed in our previous work [19] (also reproduced here); (ii) a 180°-rotated structure where P112 (*in red*) and C-terminal helix (*in orange*) face the 15LOX-1 β-barrel instead of its α2 helix (*Model 2,* Supplementary Fig. S1B); (ii-iv) two variants of *Model 1* with slightly different association sites on 15LOX-1 α2 helix (*Models 3* and *4*). Examination of the stability of these models by MD simulations of 200 ns in explicit water showed that *Models 1*–*3* were stable in solution and retained their closed form (tight interactions between PEBP1 and the α2 helix and β-barrel of 15LOX-1) as indicated by the RMSDs in atomic coordinates that remained around 3–4 Å; whereas *Model 4* exhibited three times higher RMSDs. Supplementary Fig. S2 provides a description of the simulation protocol, and Supplementary Fig. S3 displays the time evolution of RMSDs for the four systems, each conducted in triplicate, except for *Model* 4 that showed large departures from the original pose in two runs. *Model 4* was excluded from further studies due to its instability, and the conformational dynamics of *Models 1*–*3* were further investigated.

Membrane interactions trigger opening of the complex to expose a pore for phospholipid access to the catalytic site.

Several studies have shown that membrane association may elicit conformational changes in peripheral proteins, thus impacting their function [55–58]. We performed MD simulations for *Models 1*–*3*. Supplementary Table 2 describes the set of MD runs (a total of 7.7 μs) carried out for *Models 1*–*3* under different conditions (with/without membrane; with wildtype PEBP1 or its mutants, anchored to the membrane). The membrane was composed of DOPC (50%), DOPE (30%) and the most favorable 15LOX-1/PEBP1 substrate [1,12], SAPE (20%).

**Fig. 1.**
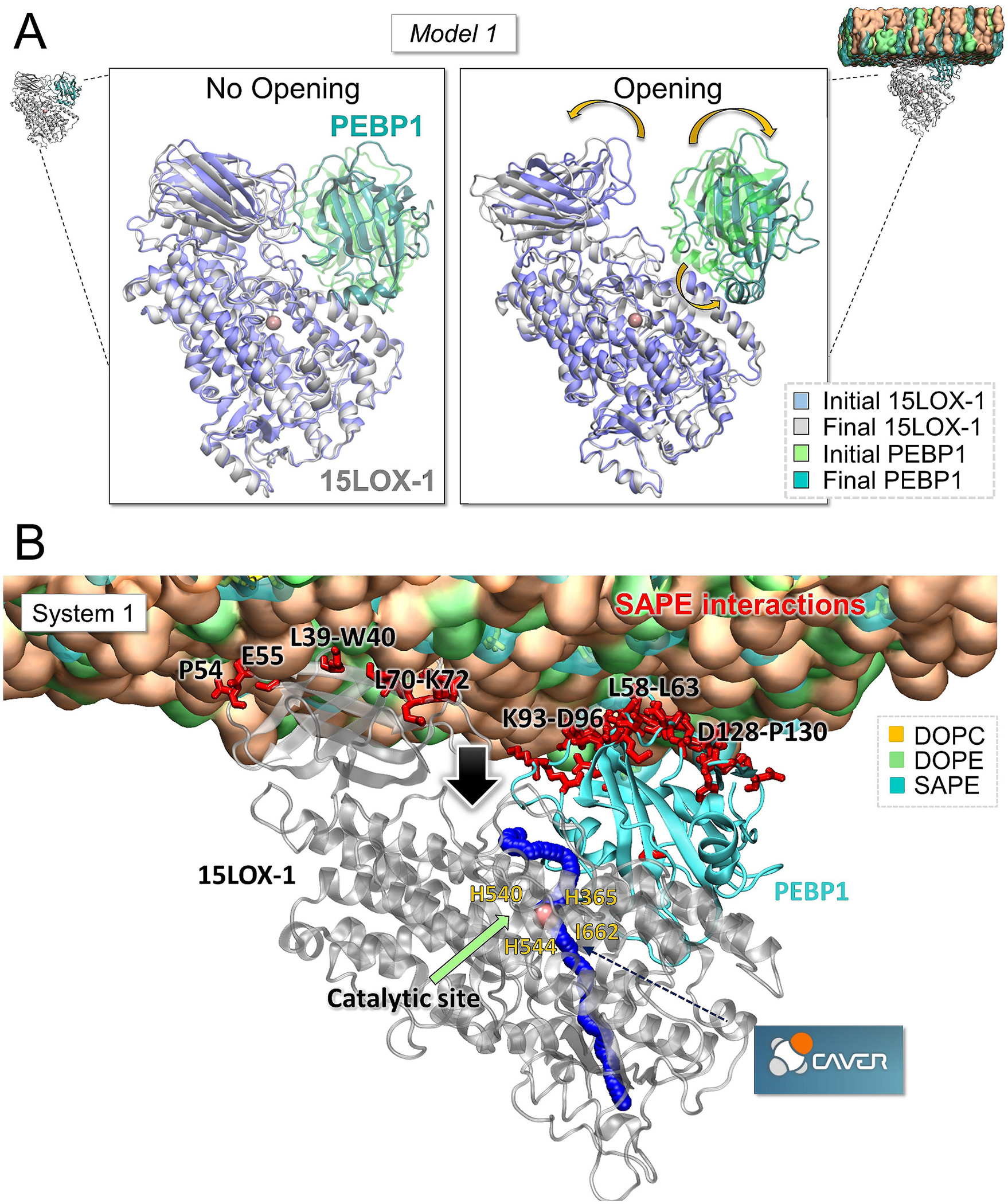
MD simulations of 15LOX-1/PEBP1 complex for *Model 1.* **(A)** Initial and final conformations of *Model 1* in the absence (*left* panel) and presence (*right* panel) of the membrane. **(B)** Closeup view of the open conformation of 15LOX-1/PEBP1 associated with the membrane (DOPC in *orange*, DOPE in *green*, and SAPE in *blue*) captured after 250-ns MD simulation. Catalytic residues (H365, H540, H544 and I662) are labeled. The *pink sphere* represents the iron in the catalytic site of 15LOX-1. Residues displayed as *red sticks* make close contacts with SAPE molecules. *Blue trace* along the 15LOX-1 structure is a visualization of the tunnel predicted by CAVER (see Methods). *Black arrow* points to the entrance of the channel for the potential insertion of SAPE molecules into the catalytic site. Results for *Model 3* can be found in Supplementary Fig. S4.

Simulations indicated that the interactions of the 15LOX-1/PEBP1 complex with the membrane promoted a conformational change that exposed the catalytic site of 15LOX-1 to the membrane, thus making the site more accessible to substrates (SAPEs) embedded in the membrane. Fig. 1 and Supplementary Fig. S4 illustrate the results for *Models 1* and *3*, respectively. This type of conformational change was not observed in the absence of membrane; whereas it was consistently reproduced in multiple runs in the presence of membrane. However, in *Model 2*, P112 was displaced by approximately 10 Å from the nearest 15LOX-1 residue. This conformational change disrupted the interactions involving P112, which have been reported to be crucial for complex formation [19]. As a result, we focused on *Models 1* and *3* for analyzing the opening mechanism. Specifically, we performed a comparative analysis of the atomic interactions at the initial and final stages of the simulations. The analysis revealed a reorganization of the interactions between 15LOX-1/PEBP1 and the membrane leading to an increase in the number of hydrogen bonds formed between the lipids and 15LOX-1 β-barrel residues N19, K37-R43, E48-E52, R68-H69 and K72-D74 and PEBP1 residues S60, D56, G61, D128, R129 and R161, compared to the initial stage of the simulation (Supplementary Fig. S5).

Next, we examined the cavities and interior surfaces of the complex in the open form, so as to identify a pore that connects the newly exposed surface and the catalytic site. Fig. 1B for *Model 1* and Supplementary Fig. S4B for *Model 3* display a *blue trace* across 15LOX-1, which also connects the exposed region to the catalytic site. We note that the entry of this predicted pore corresponds well to those previously observed for entry of AA into various LOXs, including rabbit 15LOX-1 (R403), 8(R)-LOX (R183, Y179), and soybean LOX1 (T259, L541) [59]. Alignment of these three structures within 15LOX-1 specifically highlights the significance of R402 and L178. The analysis of the interactions with the lipid molecules further indicates that membrane SAPEs were attracted by several residues, including L70-K72 (15LOX-1) and L58-K62 and R129 (PEBP1), as displayed in Supplementary Figs. S4 and S6, and Fig. 1B by *red sticks*. These residues are localized at both sides of the opening. The open space between PEBP1 and 15LOX-1 β-barrel, revealed here, is proposed to serve as an entry for SAPE molecules to reach the catalytic site of 15LOX-1 and generate the ferroptotic cell death signals, lipid hydroperoxides (15-HpETE-PE or 12-HpETE-PE).

### 3.1. Substrate binding locks the complex in a closed form

**Fig. 2.**
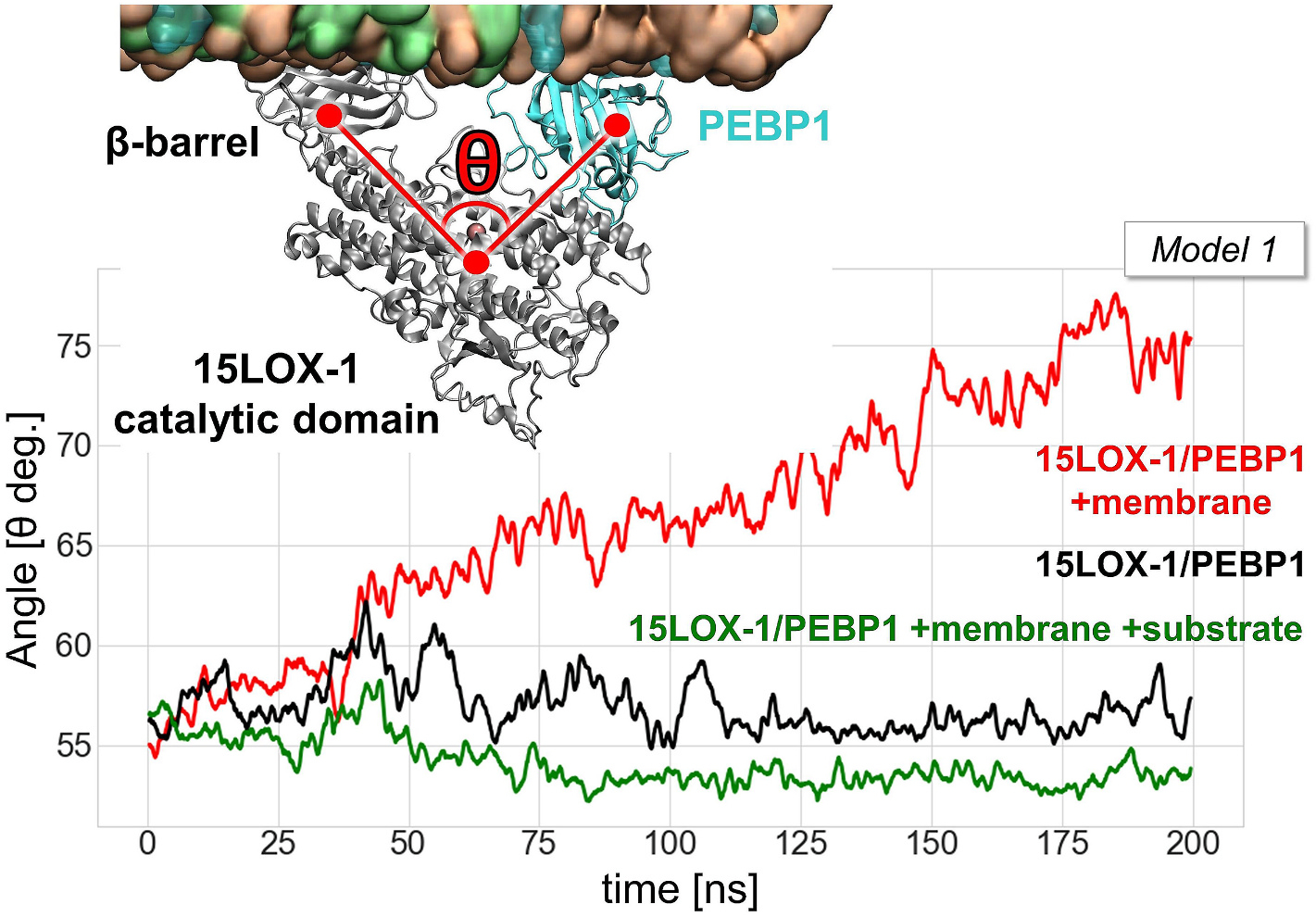
Time evolution of the angle θ in *Model 1* for the 15LOX-1/PEBP1 complex simulations with/without the membrane and in the presence of bound SAPE at the catalytic site. θ is defined as the angle between center of the mass of β-barrel, catalytic domain and PEBP1 structure as indicated by the inset.

To directly assess the ability of the membrane-embedded SAPE to approach and insert near the catalytic site of 15LOX-1, we performed MD simulations to examine the docking of SAPE onto the complex, and the stabilization of the SAPE-bound complex (Supplementary Table 2). The simulations revealed the propensity of SAPE to induce tight interactions and relatively more compact conformers upon binding to 15LOX-1/PEBP1. Fig. 2 shows the time evolution of the angle θ defined between the center of the mass of the 15LOX-1 β-barrel, its catalytic domain, and the PEBP1 mass center, as a measure of the degree of exposure of 15LOX-1 catalytic site in the complex. The angle increased by about 20° upon association of the complex with the membrane, in the absence of direct interactions with SAPE molecules (*red curve* in Fig. 2 for *Model 1*; see also the data from multiple runs in Supplementary Fig. S7). In contrast, in the case of membrane- and substrate-bound complex, θ decreased by about ~5° (*green curve*, Fig. 2).

The opening/closure of the catalytic site in 15LOX-1 was found to be strictly dependent on its occupancy. In the open form, the complex facilitated the diffusion of the substrate, while substrate binding led to the closure and stabilization of the catalytic pocket, enabling the oxidation reaction. SAPE-bound *Model 3* generally exhibited a lower binding affinity (−12.62 ± 0.15 kcal/mol) compared to *Model 1* (−14.99 ± 0.28 kcal/mol), and it displayed a potentially unreactive orientation with respect to the iron at the catalytic site (Supplementary Fig. S8).

### 3.2. Key residues stabilize the 15LOX-1/PEBP1 complex and enable its association with SAPE

Statistical analysis of interfacial interaction between 15LOX-1 and PEBP1 revealed several critical contacts (Supplementary Fig. S9). Residues engaged in stable interactions include D169-K170, E175-V176, A179-I187, R402, L157-L160, and D410-M411 on 15LOX-1, and K80-Y81, R141-H145, K148, with P112-K113 and C-terminal helix residues (Y181-E182, S185-G186) on PEBP1, consistent with those reported to be critical to complex formation [19].

**Fig. 3.**
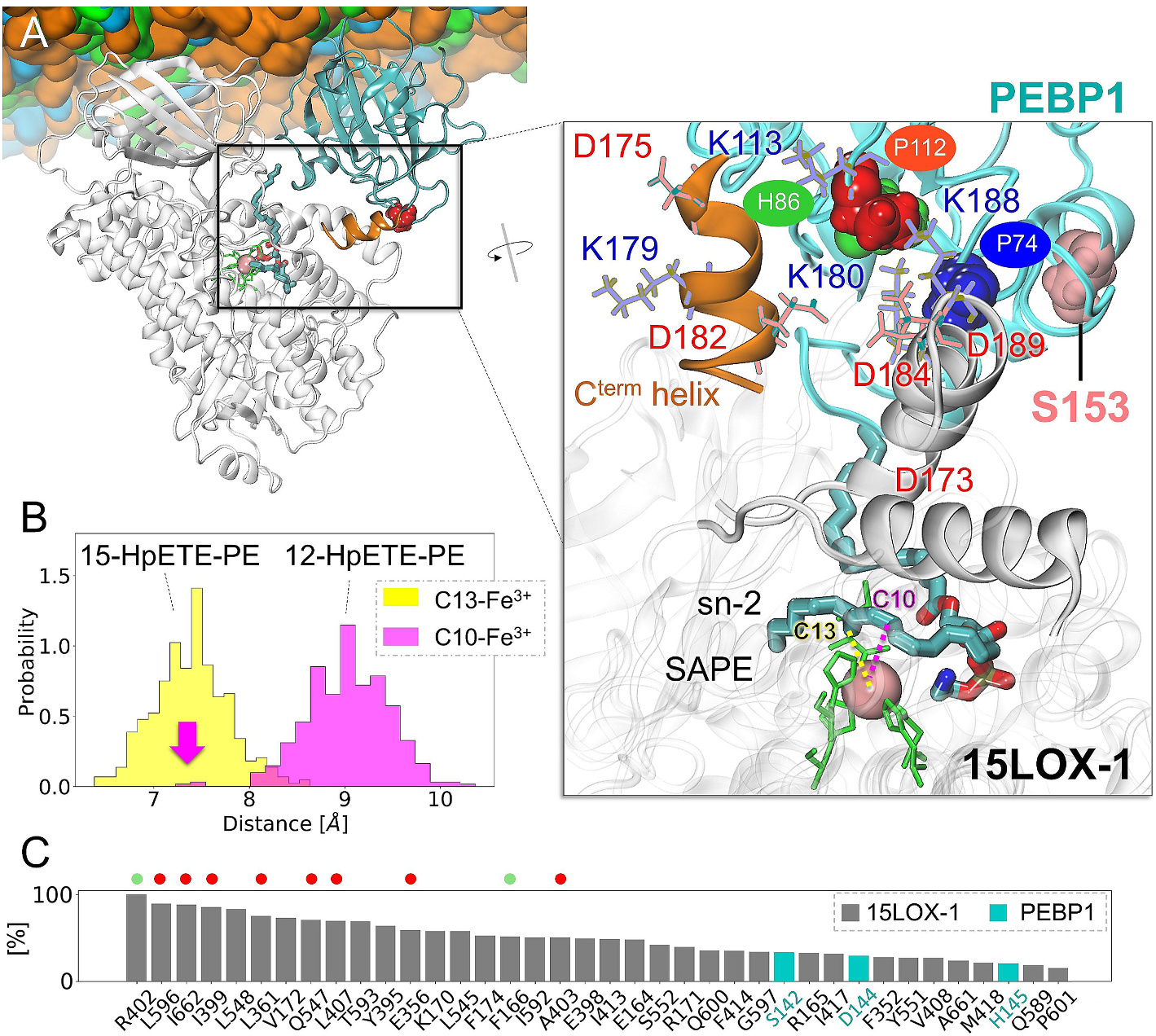
Interactions between 15LOX-1/PEBP1 and SAPE. **(A)** 15LOX-1/PEBP1/SAPE complex in *Model 1* after 200 ns MD simulation with the membrane. The *inset* shows a close view of the SAPE-binding interface of 15LOX-1/PEBP1. The C-terminal helix (D175-Y186) is shown in *orange*; *red, blue, green*, and *pink spheres* refer to P112, P74, H86 and S153 of PEBP1, respectively. The catalytic residues H365, H540, H544 and I662 are displayed in *green sticks*. All charged residues within 7 Å from P112 are displayed. **(B)** Probability distribution of the distance between iron and hydrogen donors (C13 (*yellow histogram*) and C10 carbons (*pink histogram*)) of SAPE which correspond to the 15-HpETE-PE and 12-HpETE-PE products. *Pink arrow* refers to the occurrence of C10-iron distance at ~7.2 Å. **(C)** Distribution of the most frequent contacts between SAPE and 15LOX-1/PEBP1. The count numbers are normalized.

In the presence of substrate, the interactions of 15LOX-1 D410 were replaced by those of R598-R599 due to the reconfiguration of the 15LOX-1/PEBP1 complex into a closed conformer. This reconfiguration, observed in *Model 1*, was stabilized after ~50 ns simulation (*green curve*, Fig. 2) and tightened upon binding a SAPE molecule. SAPE made stable contacts with several residues highly conserved among LOX family members (*red dots*, Fig. 3C), e.g., leucines (L596, L407, L361). The counterparts of these leucines in 15LOX-2 (L610, L420 and L374, respectively) have been identified to play a critical role in 15LOX-2/SAPE interactions [33]. Moreover, 15LOX-1 R402 and F166 (highlighted as *green dots*, Fig. 3C) occupy a central hinge region of LOX, shared between mammalian and bacterial forms, but not at the same sequential position [5]. The presence of arginine at this specific location has been documented in various studies on LOXs, including 12LOX (R403 [60]), 15LOXs (R402 [5]), PA-LOX (R422 [5]), or 8(R)-LOX (R182 [61]), not only as an entry to the catalytic site but also in relation to substrate interactions. Finally, we notice that PEBP1 residues S142, D144, and H145 are engaged in close interactions with SAPE (Fig. 3C). This region may be critical for PEBP1 dimerization [62] triggered by PKC-mediated phosphorylation of S153 and has been considered as an important mechanistic feature of PEBP1 substrate specificity [62,63]. Our model, shown in Fig. 3A (*inset*), indicates the accessibility of S153 (*pink sphere*) to a potential modification that may trigger complex dissociation.

**Fig. 4.**
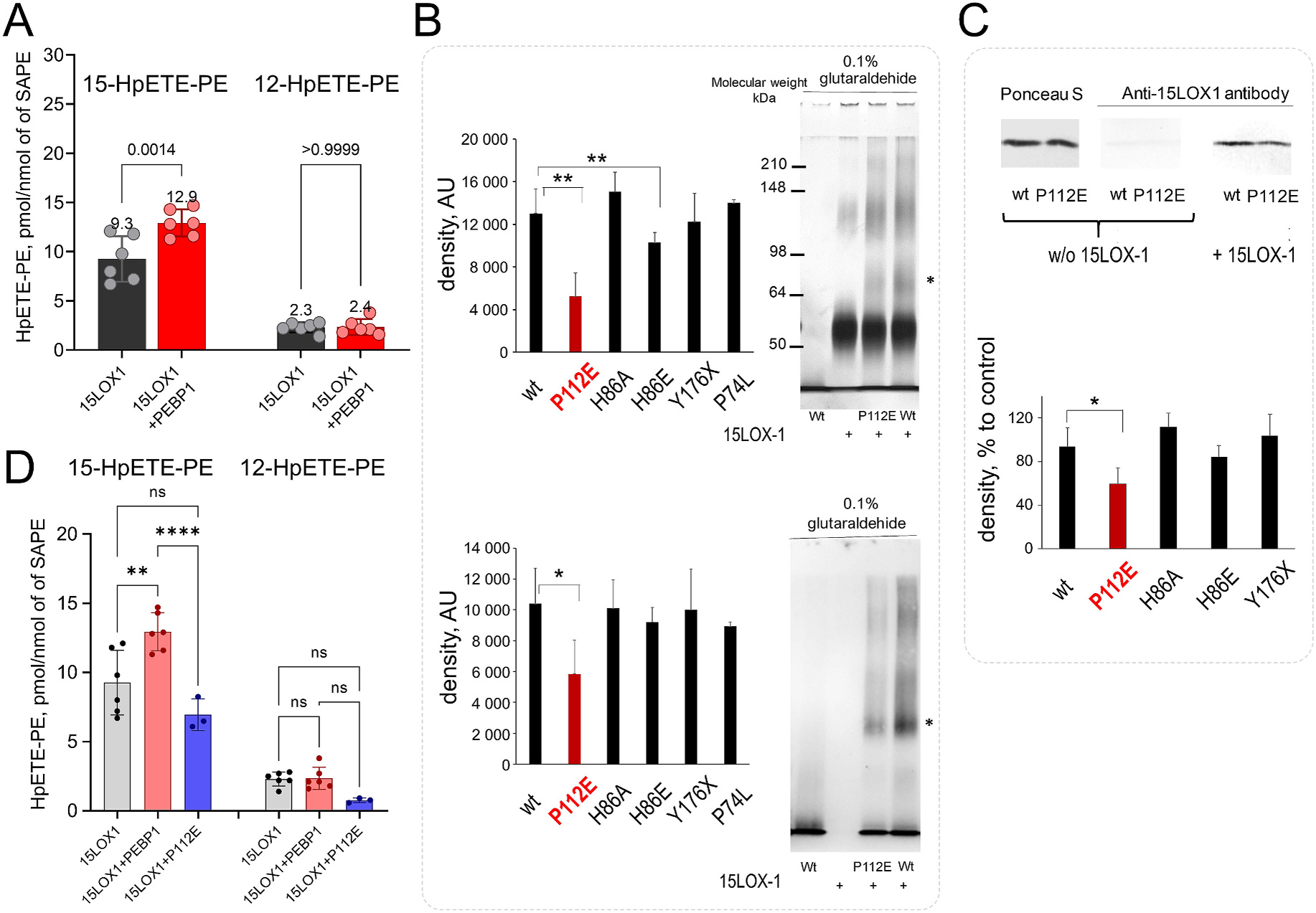
Mutations in PEBP1 and their effects observed by LC-MS. **(A)** *Bar plot* comparing 15-HpETE-PE and 12-HpETE-PE formation by 15LOX-1 and by 15LOX-1/PEBP1. Data are mean ± s.d., n = 6 (ANOVA test). SAPE:DOPC liposomes were incubated with 0.04 μM 15LOX-1±PEBP1 (1:1) for 2.5 min. **(B)** Interaction of 15LOX-1 with wild type and mutated forms of PEBP1. Samples containing 15LOX-1 and PEBP1 were treated with 0.1% glutaraldehyde for 20 min at room temperature and reaction was stopped by the addition 200 mM of Tris –HCl (pH 7.5). Samples were run in SDS-PAGE and in some cases, electro-transferred to nitrocellulose membrane. Proteins were revealed by staining with silver (*upper panel*) or by incubation with anti–PEBP1 antibodies (*lower panel*). The density of the band that appeared after incubation of PEBP with 15LOX-1 in the presence of glutaraldehyde (marked by asterisks) was assessed to be significant only in the presence of P112E and H86A mutants.**P *<* 0.001, *P < 0.05. **(C)** Binding of 15LOX-1 to wt PEBP1 and PEBP1 mutants in the presence of dioleoyl-PE containing liposomes revealed by Far Western blotting. Ponceau S staining of proteins (*left panel*). Immuno-detection of 15LOX-1 bound to PEBP1 using anti-15LOX-1 antibody (*right panel*). Membrane containing electro-transferred PEBP1, but not incubated with 15LOX-1, was used as a control (*central panel*).*P < 0.05. Quantitation of 15LOX-1 bound to wt PEBP1 and mutant forms of PEBP1 with anti-15LOX-1 antibody. **(D)** *Bar plot* comparing 15-HpETE-PE and 12-HpETE-PE formation by 15LOX-1, 15LOX-1/PEBP1, and by 15LOX-1/P112E PEBP1. SAPE:DOPC (1:1, 100 μM) liposomes were incubated with 0.04 μM 15LOX-1 ± PEBP1 (1:1) for 2.5 min or 0.04 μM 15LOX-1 ± PEBP1 mutant P112 (1:1) for 5 min. Data are mean ± s.d., One-way ANOVA.

Both 15LOX-1 and 15LOX-2 catalyze the oxidation of SAPE by abstracting a hydrogen at carbon atom C13 and inserting an oxygen atom at C15 resulting in the 15-HpETE-PE product. The isoform studied here is also able to occasionally abstract a hydrogen at the C10 atom (ratio 1:9 for arachidonic acid as substrate [25,64]) to generate an alternative product, 12-HpETE-PE. To determine whether one or both products could be produced according to our computational model, we checked the distance between the hydrogen donors (C13 and C10) and the iron at the catalytic site (Fig. 3A and B). The results showed that the iron generally maintains a shorter distance from C13 (mean distance: ~7.2 Å, *yellow histogram*) compared to C10 (~9 Å, *magenta histogram*). We detected only a few cases where C10 was at a close distance of ~7.2 Å (*pink arrow*). The lower probability of C10 to be close to the iron at the catalytic site, compared to C13, is in agreement with the experimentally observed (Fig. 4A, D) lower production of 12-HpETE-PE (ratio 1:5 for 15LOX-1/PEBP1 complex), compared to 15HpETE-PE. Moreover, the production of 12-HpETE-PE, in contrast to 15-HpETE-PE, was not affected by the presence of PEBP1.

### 3.3. Experiments demonstrate the critical role of P112 among the interfacial residues of PEBP1

Next, we used two different protocols to experimentally assess PEBP1 binding interactions with 15LOX-1: i) crosslinking by an amine-directed reagent, glutaric dialdehyde and ii) Far Western blotting [65]. Results demonstrated that treatment of the proteins with glutaraldehyde leads to the appearance of additional band detectable by silver staining and anti-PEBP1 antibody. To assess the role of different amino acids in PEBP1/15LOX-1 association, we prepared several mutants of PEBP1, such as P112E, H86A, H86E, Y176X and P74L. Complex formation was strongly suppressed when P112E mutant was used, while the other mutations did not affect the PEBP1/15LOX-1 complex formation (Fig. 4B). In the Far Western blotting protocol, an antibody-positive “bait” protein (15LOX-1) is used to detect the target “prey” protein (PEBP1). The bands corresponding to PEBP1 were also 15LOX-1-positive (Fig. 4C). These experiments also showed that the formation of PEBP1/15LOX-1 complex was strongly suppressed when P112E was used in place of wt PEBP1. In contrast, the interactions of the other PEBP1 mutants with 15LOX-1 were similar to that of the wt protein.

P112E mutation also inhibited the production of both 15-HpETE-PE and 12-HpETE-PE by 15LOX-1/PEBP1 (Fig. 4D). This was particularly obvious for 15-HpETE-PE whose formation was completely abrogated by the P112 mutation (compare Fig. 4A and D).

**Fig. 5.**
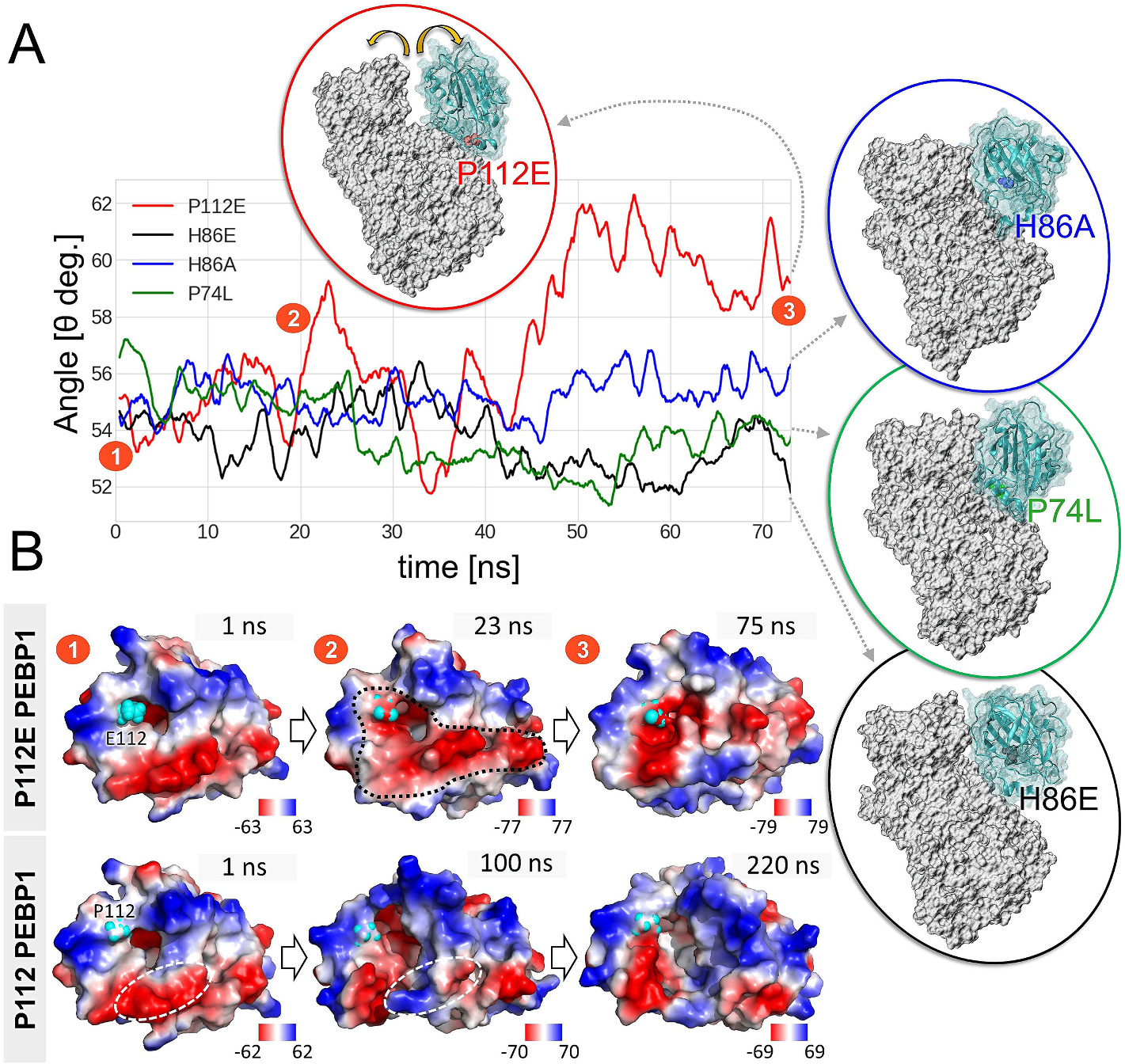
Mutations at PEBP1 interfacial residues and their effects observed in simulations. **(A)** Time evolution of the angle θ for the complex of 15LOX-1/SAPE and four types of PEBP1 mutants, P112E, H86A, H86E, and P74L. The definition of the angle θ together with the wt PEBP1 results can be found in Fig. 2. *Yellow arrows* indicate a significant conformational change, which appears in the complex with P112E mutant. Displayed complexes are the final conformations from 75 ns of MD simulations. **(B)** Electrostatic surface potentials obtained from APBS-PDB2PQR software suite for wt PEBP1 and mutant P112E conformations from the simulations (0–220 ns for wt and 0–75 ns for P112E mutant). Positively charged regions are shown in *blue*, and negatively charged regions in *red*. The energy values are in units of kT/e.

To computationally examine the validity of the predicted model, we next considered the experimentally analyzed mutations, H86A, H86E, P74L and P112E (Fig. 4) on PEBP1 and their potential effect on the complex formation. Therefore, we prepared four types of 15LOX-1/PEBP1/SAPE systems, each corresponding to a different mutant of PEBP1, and performed 75 ns MD simulations in the presence of the membrane (Fig. 5). The results indicate that the mutations H86A, H86E and P74L were neutral (Fig. 5A, *blue, green,* and *black* insets). The corresponding complexes were stable and angle θ defined above exhibited the same behavior as that observed for the wt PEBP1 in its complex with 15LOX-1/SAPE (Fig. 2 *vs blue, green* and *black* curves in Fig. 5A). In contrast, the P112E mutation caused a significant conformational change in the PEBP1/15LOX-1/SAPE system that triggered the opening of the complex after ~20 ns (Fig. 5A, *red* curve, #2, Movie 1). This reorganization led to a short period of more favorable interactions at a slightly different 15LOX-1 region (30–40 ns), but finally resulted in a wide opening of the complex (>45 ns; *red inset*, #3).

During this period, the electrostatic potential at P112E changed drastically compared to that of the wt PEBP1 (Fig. 5B). Initially, the negatively charged regions, similar for both wt and mutant P112E PEBP1, were centered on D134-E135, S142-D144, E159, and E182-G186 (*dark red*, #1). However, after 20–24 ns, the potential energy increased by over 20% (from −63 to −77 kT/e, #2) and additional residues, such as D69, D72, G108-S109, P112E, D144-H155, contributed to a highly negative potential energy which resulted in the repulsion of the α2 helix of 15LOX-1 (see Movie 1). This effect was coupled to a loss of interactions near a hub residue E175 (on 15LOX-1, *pink sphere* in Movie 1) with PEBP1 K148, Y81 and W84 (*thin blue, green and white sticks*), thus leading to E175-K113 interaction instead, and further reorganization in electrostatic potential in P112E PEBP1 (#3). During the simulations, we observed slight changes in the electrostatic potential of the wt PEBP1 at the interface with 15LOX-1. Originally, the interface was negatively charged, indicated by a *white dashed oval* at 1 ns; then it underwent a transition to a more neutral and positive state upon complexation with 15LOX-1, particularly near R141 and S142. This transition facilitated multiple interactions between E175 and wt PEBP1, as depicted by a *pink sphere* in Movie 2. Furthermore, negatively charged regions formed on both sides of the α2 helix of 15LOX-1 (P112 mutant >100 ns) centered around G108-S109, Y181-G186, and D134-E135. Those changes, however, did not affect PEBP1/15LOX-1 interactions which were constantly maintained for over 100 ns. Additionally, we noticed that the loss of interactions between P112E mutant and 15LOX-1 (Movie 1) not only initiated the opening of the complex, but might also have impacted the catalytic activity of the enzyme by destabilization of the E164-F174 loop that interacts with the catalytic I662 through K170 (Movie 1, *thick blue sticks*). This loop maintained stable interactions in wt PEBP1 system.

## 4. Discussion

Ferroptosis is an iron-dependent cell death driven by the production of PUFA-PE hydroperoxides which serve as ferroptotic death signals [19]. Our recent work revealed that Fe-containing 15-LOX plays a major role in catalyzing the peroxidation of PUFA-PE, and in particular SAPE; and this catalytic activity is endowed upon complexation of 15-LOX with PEBP1 which modulates the substrate specificity of 15-LOX [12,19,31, 33]. We demonstrated that the anti-ferroptotic action of the most commonly used ferroptosis inhibitor ferrostatin-1 is not limited to radical scavenging but also includes the suppression of SAPE peroxidation by 15LOX/PEBP1 [30]. Furthermore, we recently proposed two compounds that effectively suppress ferroptosis *in vitro* and *in vivo* upon targeting the interactions with 15LOX-2/PEBP1 [66]. These studies consistently highlight the pivotal role of PEBP1 in ferroptotic events. With this in mind and given the still largely enigmatic nature of enzymatic peroxidation, we conducted here a first computational study of the membrane-bound 15LOX-1/PEBP1/SAPE system dynamics, and showed that the computational predictions are consistent with previous [19] and current experimental data. The study helps advance our understanding of the molecular mechanisms underlying this crucial form of cell death.

Our study shows that the membrane plays a regulatory role in 15LOX/PEBP1 catalytic activity by inducing a conformational change that exposes an entry site conducing to 15LOX catalytic pocket, thus allowing SAPE molecules to access the catalytic site (Fig. 1). Additionally, the binding of SAPE not only stabilizes but also tightly locks the complex (Fig. 2), leading to an optimal pose for the peroxidation reaction (Fig. 3). Membrane interactions are known to trigger the activation of peripheral proteins, including those strictly associated with PL oxidation and the hydrolysis of PL oxidized forms (e.g., cytochromes [35–37] or phospholipases [10,38]). The current study provides another example of the important regulatory role of the lipid bilayer, this time in facilitating the catalytic activity of 15LOX in the 15LOX-1/PEBP1 complex to generate pro-ferroptotic oxidized PEs.

Our computational findings are at many levels consistent with the experimental data (Fig. 4A). The complex can generate two distinct peroxidation products, 15-HpETE-PE and 12-HpETE-PE. Among them, the former is PEBP1-dependent as indicated by LC-MS data and conformed by computations (Fig. 3A and B). We identified key residues involved in stabilizing the 15LOX-1/PEBP1 complex (Supplementary Fig. S9) and in the interactions with SAPE (Fig. 3C). Evaluations of the predicted 15LOX-1/PEBP1 interactions with PEBP1 mutations at residues participating in interfacial interactions with 15LOX-1, both *in silico* and in experiments (Fig. 4B–D and 5) revealed the key role of P112. Among five such mutations (H86A, H86E, P74L, Y176X and P112E), P112E was distinguished by its effect on both 15LOX-1/PEBP1 complex formation (Fig. 4B and C, Fig. 5) and peroxidation mechanism (Fig. 4D).

Overall, the present study provides new insights into the molecular basis of the formation of the enzymatic complex 15LOX-1/PEBP1, the modulation of 15LOX catalytic activity by both PEBP1 and membrane lipid molecules themselves, and the access of the substrate, SAPE, into the catalytic pocket of 15LOX. The study highlights the significance of the localization of 15LOX/PEBP1 at the membrane periphery for the occurrence of membrane-induced conformational changes thus predispose 15LOX to catalyze the hydroperoxidation of PUFA-PEs. We further provide a plausible mechanism for PUFA-PE binding and insertion into the catalytic pocket of 15LOX, thus elucidating the way 15LOX-1/PEBP1 complex acquires lipids from the membrane to the catalytic site, a critical step in the production of peroxidized PUFA-PEs, the accumulation of which leads to ferroptosis. These findings, including those validated by experiments, holds significant potential for designing novel therapeutics that selectively target 15LOX-1/PEBP1 complexes for modulating ferroptotic events.

## Supplementary Material

supl

movies
