# Supplementary material for: Membrane regulation of 15LOX-1/PEBP1 complex prompts the generation of ferroptotic signals, oxygenated PEs
Source: Free Radic Biol Med. Author manuscript; Available in PMC 2024 Nov 1. (PMC10952060; doi:10.1016/j.freeradbiomed.2023.09.001)
Supplement: supl [file NIHMS1972082-supplement-supl.pdf]

Supplementary Material for

Membrane Regulation of 15LOX-1/PEBP1 Complex Prompts the Generation of Ferroptotic Signals, Oxygenated PEs

Thiliban Manivarma[a], Aleksandr A. Kapralov[b], Svetlana N. Samovich[b], Yulia Y. Tyurina[b], Vladimir A. Tyurin[b], Andy P. VanDemark[c], Wieslaw Nowak[a], Hülya Bayır[b,d], Ivet Bahar[e*], Valerian E. Kagan[b, f, g, h*], Karolina Mikulska-Ruminska[a*]

[a] Institute of Physics, Faculty of Physics, Astronomy and Informatics, Nicolaus Copernicus University in Torun, PL87100 Torun, Poland.
[b] Department of Environmental and Occupational Health and Center for Free Radical and Antioxidant Health University of Pittsburgh, Pittsburgh, Pennsylvania 15260, United States.
[c] Department of Biological Sciences, University of Pittsburgh, Pittsburgh, PA, USA.
[d] Department of Pediatrics, Division of Critical Care and Hospital Medicine, Redox Health Center, Vagelos College of Physicians and Surgeons, Columbia University Irving Medical Center, New York, NY, USA
[e] Laufer Center for Physical and Quantitative Biology and Department of Biochemistry and Cell Biology, Stony Brook University, New York 11794, USA.
[f] Department of Radiation Oncology, [g] Department of Chemistry, and [h] Department of Pharmacology and Chemical Biology, University of Pittsburgh, Pittsburgh, Pennsylvania 15260, USA.

**corresponding authors: karolamik@fizyka.umk.pl (K.M.-R.), kagan@pitt.edu (V.E.K.), bahar@laufercenter.org (I.B.)*

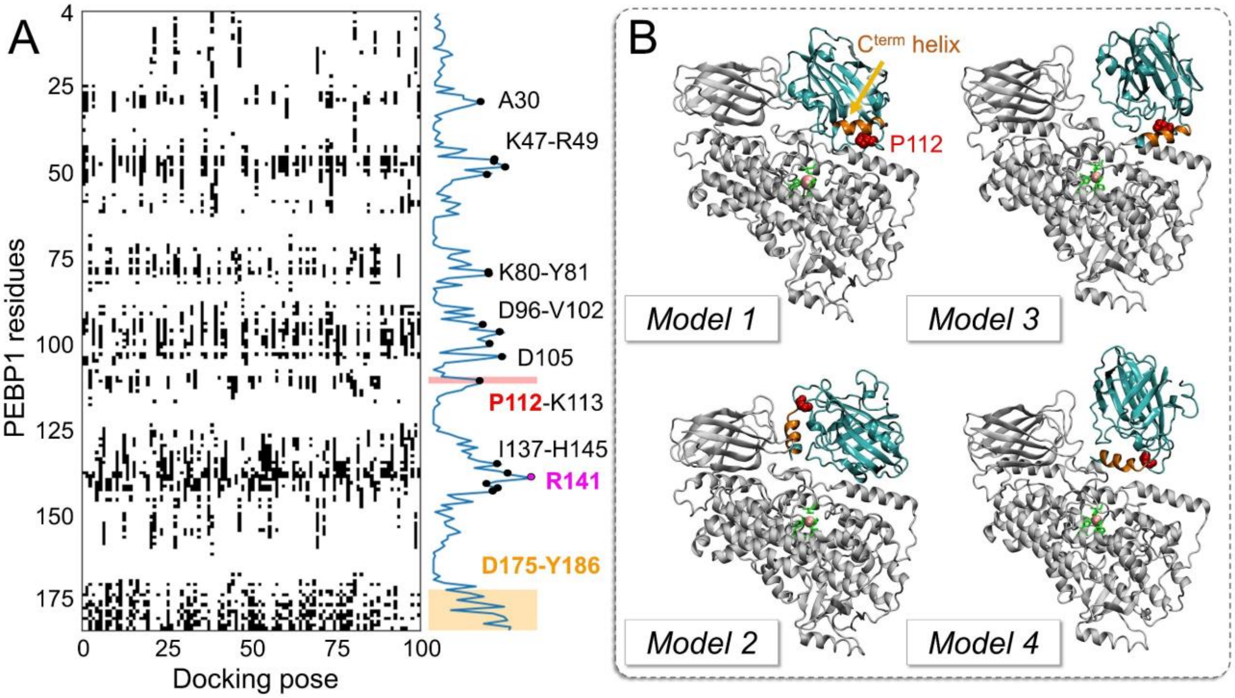

Supplementary Figure S1. Structural models for the human 15LOX-1/PEBP1 complex predicted by molecular docking simulations. (A) Detailed plot of PEBP1 interactions with 15LOX-1 observed in more than 100 docking runs (*abscissa*). The *black dots* indicate the 15LOX-1-binding residues of PEBP1 (*ordinate*) observed in each run of docking simulations. The total number of interactions per PEBP1 residues is shown as a *blue* line along the *right ordinate*. Entropically the most favorable sites, i.e. residues which make interfacial interactions in at least 30% of the runs, are labeled. P112 (*in red*) and C-terminal helix of PEBP1 (*in orange*) are experimentally confirmed sites for 15LOX-1/PEBP1 interactions. **(B)** Four conformers that exhibit interfacial contacts in line with experimental data. PEBP1 (*cyan ribbon diagram*) tends to bind at the α2 helix of 15LOX-1. 15LOX-1 is colored by the distribution of interactions with PEBP1. The residues exhibiting the highest propensity of interactions are colored *red*, those with intermediate propensity are in *white*, and those exhibiting low propensity, are in *blue*. *Orange* region corresponds to the C-terminal helix (D175-Y186) and *red sphere* to P112.

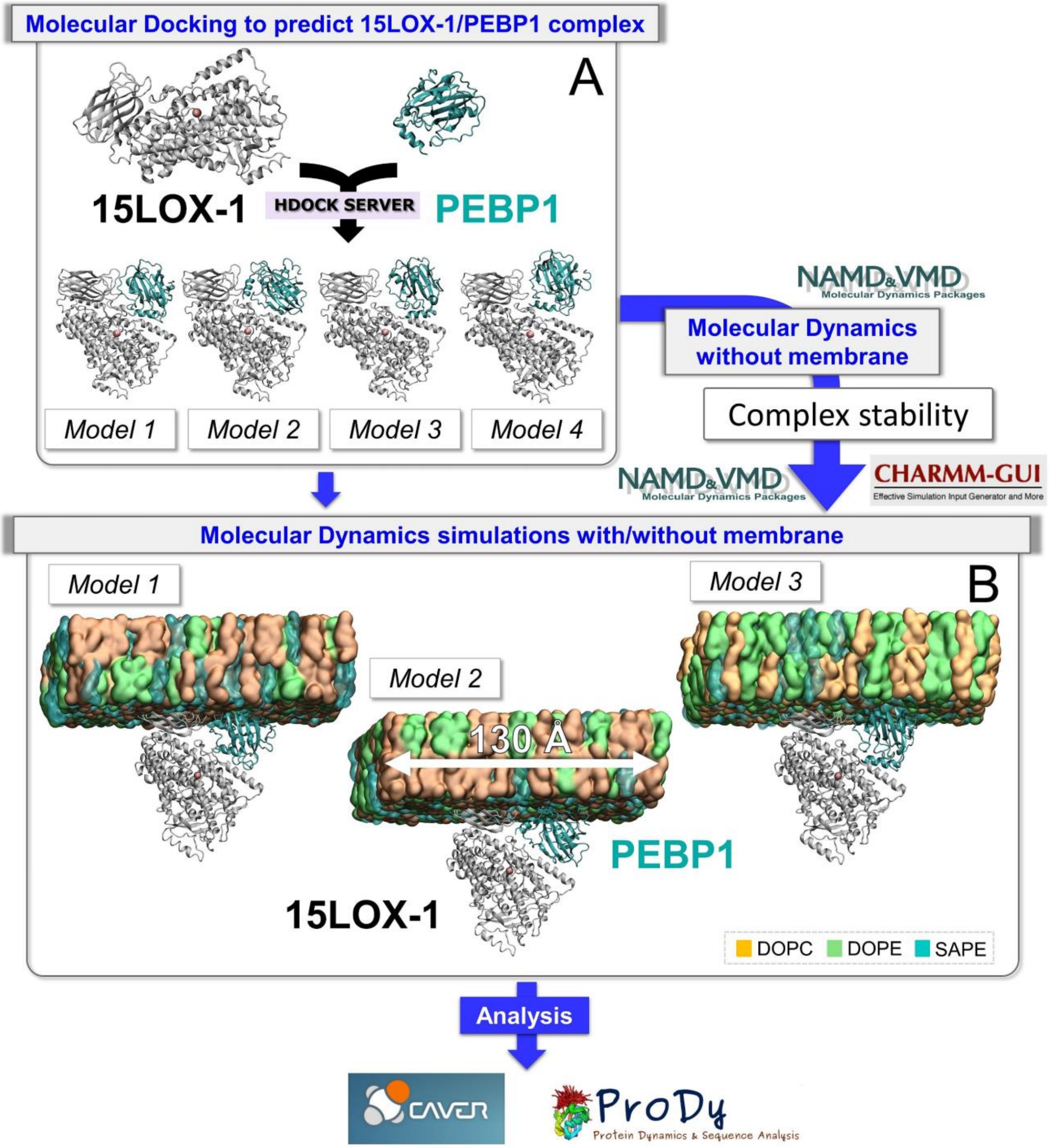

Supplementary Figure S2. Schematic description of the computational protocol adopted in the present study.

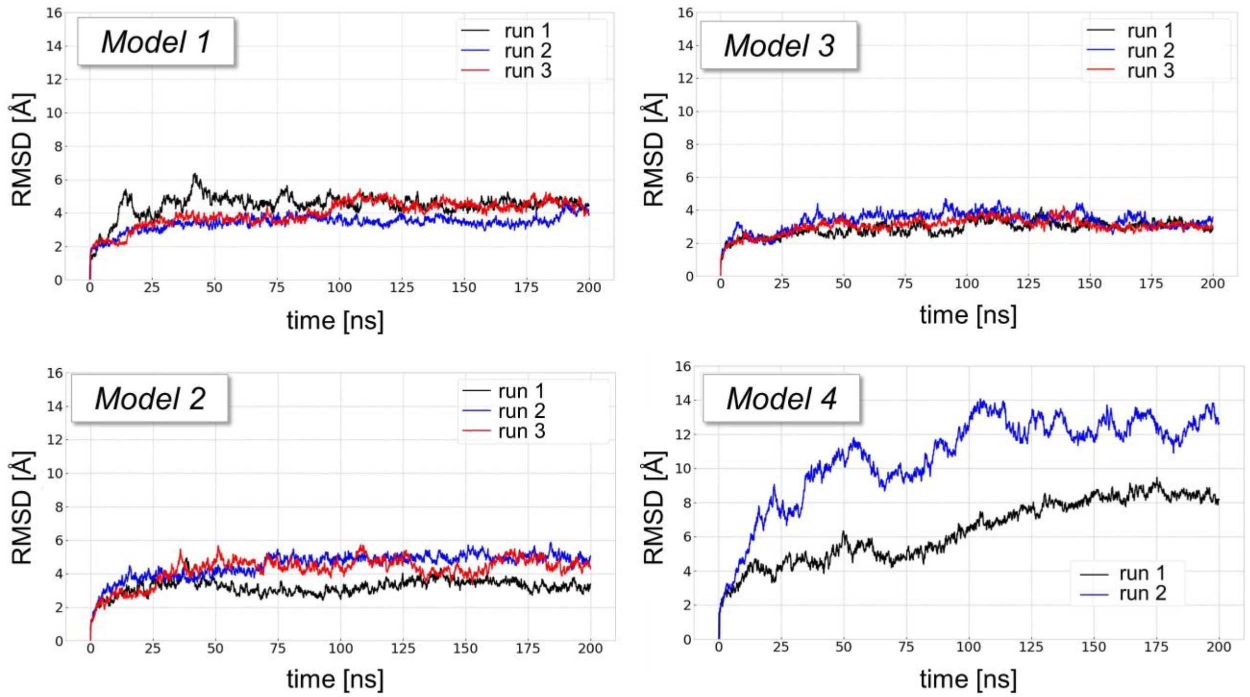

Supplementary Figure S3. RMSD in atomic coordinates relative to the starting conformer, observed for *Models 1-4* in MD simulations carried out in explicit water.

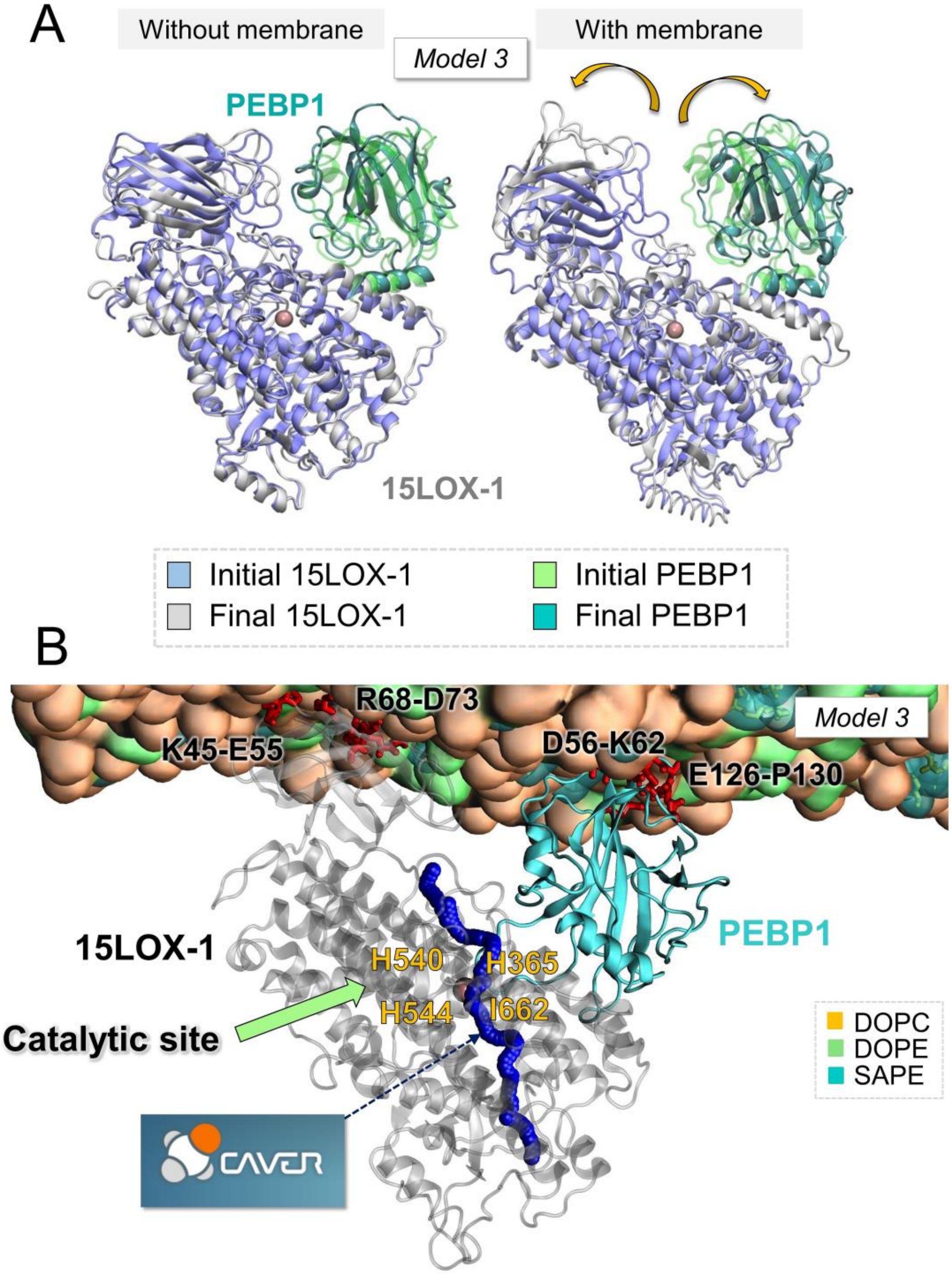

Supplementary Figure S4. MD simulations of 15LOX-1/PEBP1 complex in the presence and absence of the membrane for *Model 3*. (A) Initial and final conformations of *Model 3* in the absence (*left* panel) and presence (*right* panel) of the membrane. **(B)** Open conformation of the 15LOX-1/PEBP1 complex associated with the membrane (DOPC in orange, DOPE in green, and SAPE in blue) captured after 250-ns MD simulation. Catalytic residues (H365, H540, H544 and I662) are labeled. Pink sphere denotes the iron in the catalytic site of 15LOX-1. Residues displayed as *red sticks* make close contacts with SAPE lipids. Blue trace along the 15LOX-1 structure is a visualization of the tunnel predicted by CAVER.

Green arrow indicates the entrance of the potential channel for SAPE lipids to the catalytic site of 15LOX-1.

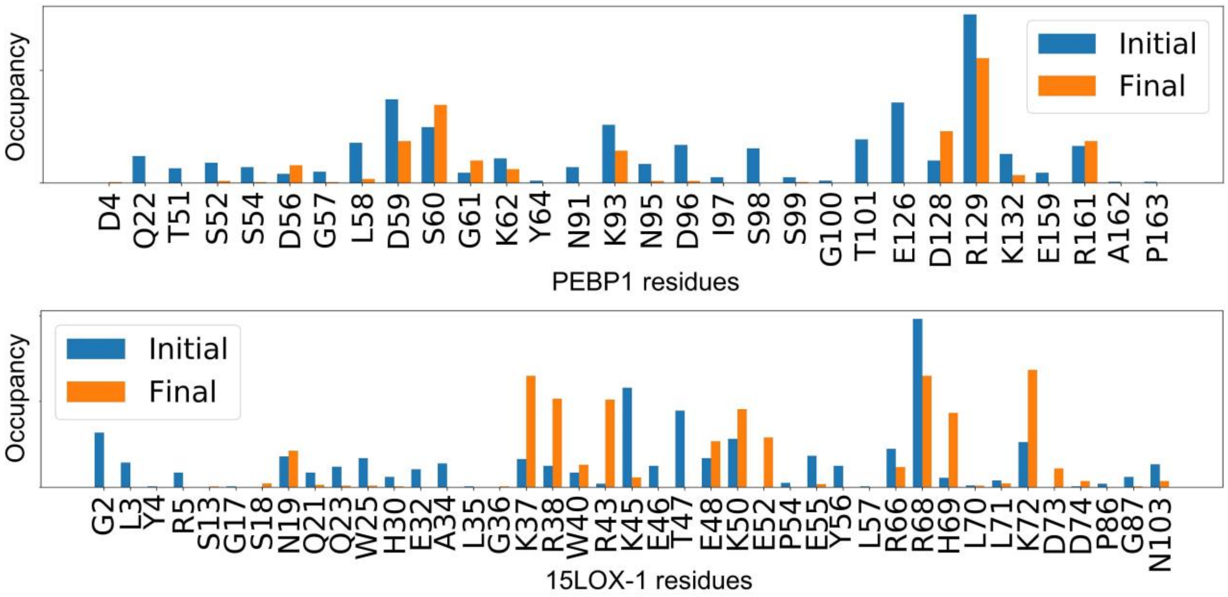

Supplementary Figure S5. Comparison of the number of hydrogen bonds formed between membrane components and the complex in first and last 20 ns of all MD simulations, i.e. before and after opening of the complex, respectively.

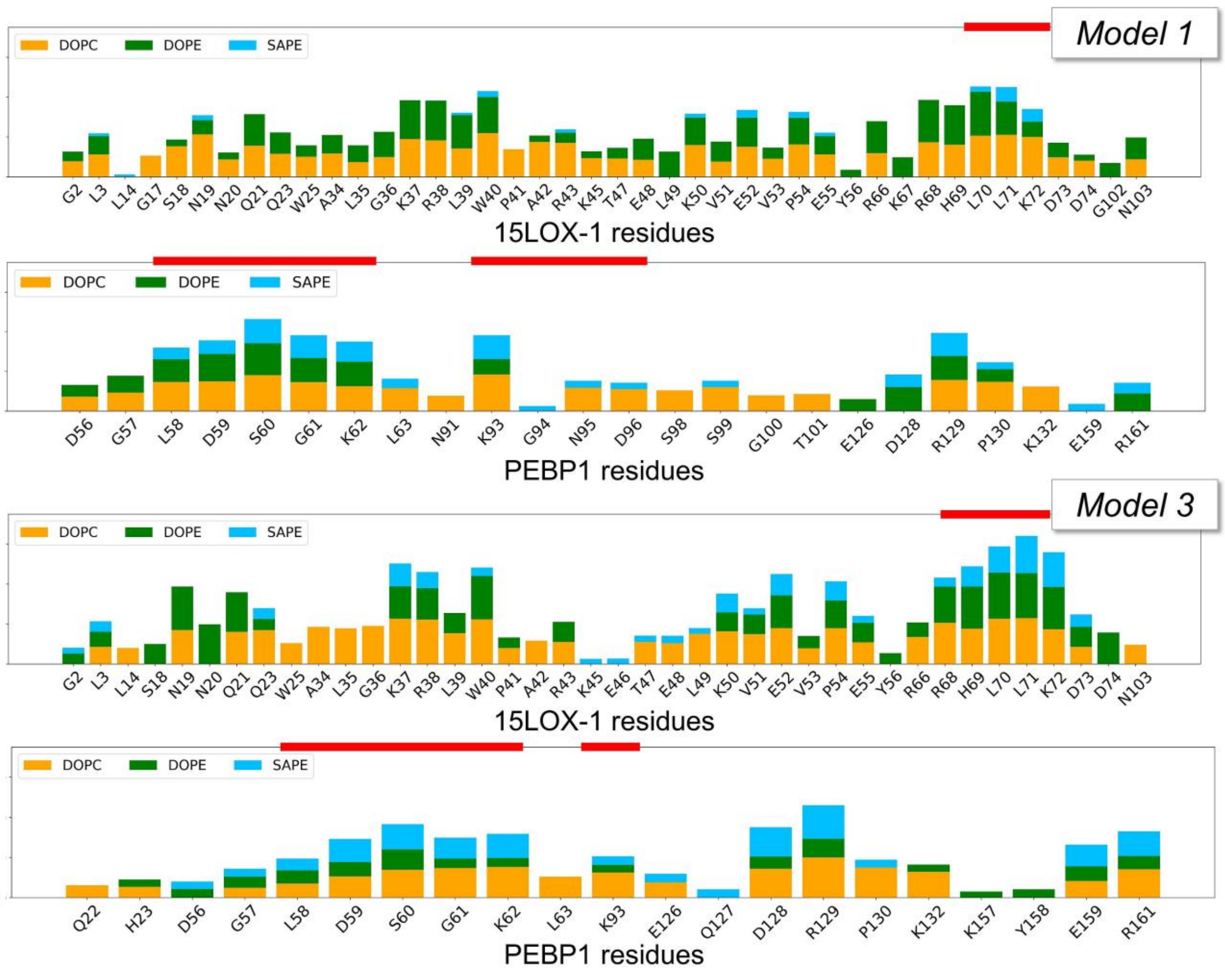

Supplementary Figure S6. Membrane binding residues of 15LOX-1 and PEBP1 for Model 1 and 3. Interfacial contacts (within 5Å) between the complex and the different types of lipid molecules (DOPC in *orange*, DOPE in *green*, and SAPE in *blue*) observed during three MD simulations (of 250 ns each). Red lines along the upper abscissa refer to the regions newly exposed upon opening of the complex.

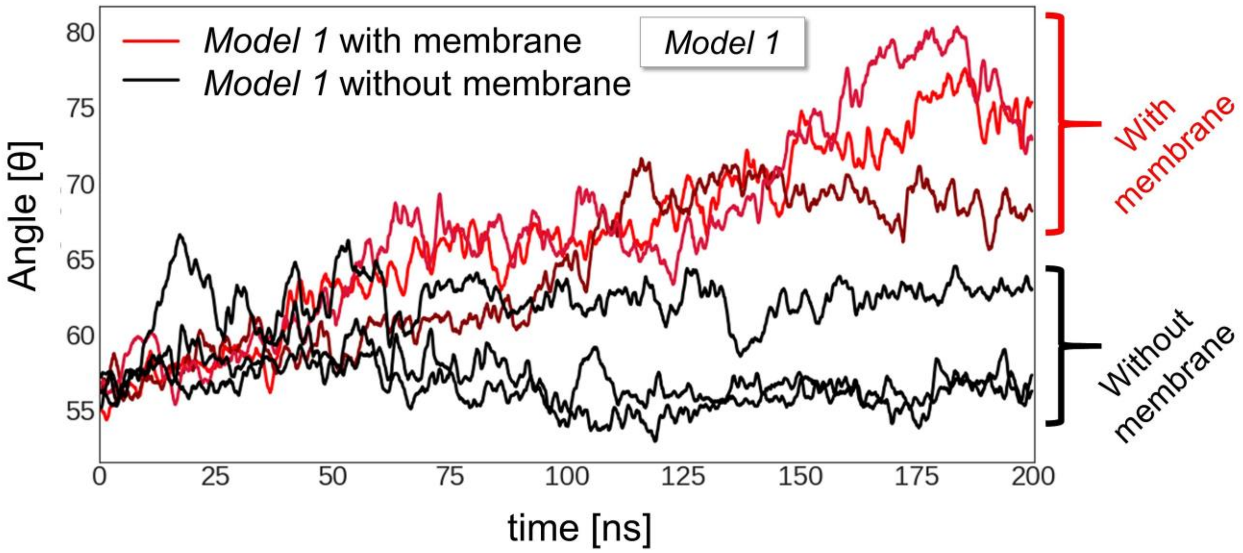

Supplementary Figure S7. Time evolution of the angle θ in *Model 1* for the 15LOX-1/PEBP1 complex in multiple independent MD runs with/without the membrane. Angle θ is defined as the angle between center of the mass of β-barrel, catalytic domain and PEBP1 structure as shown in **Fig. 2.**

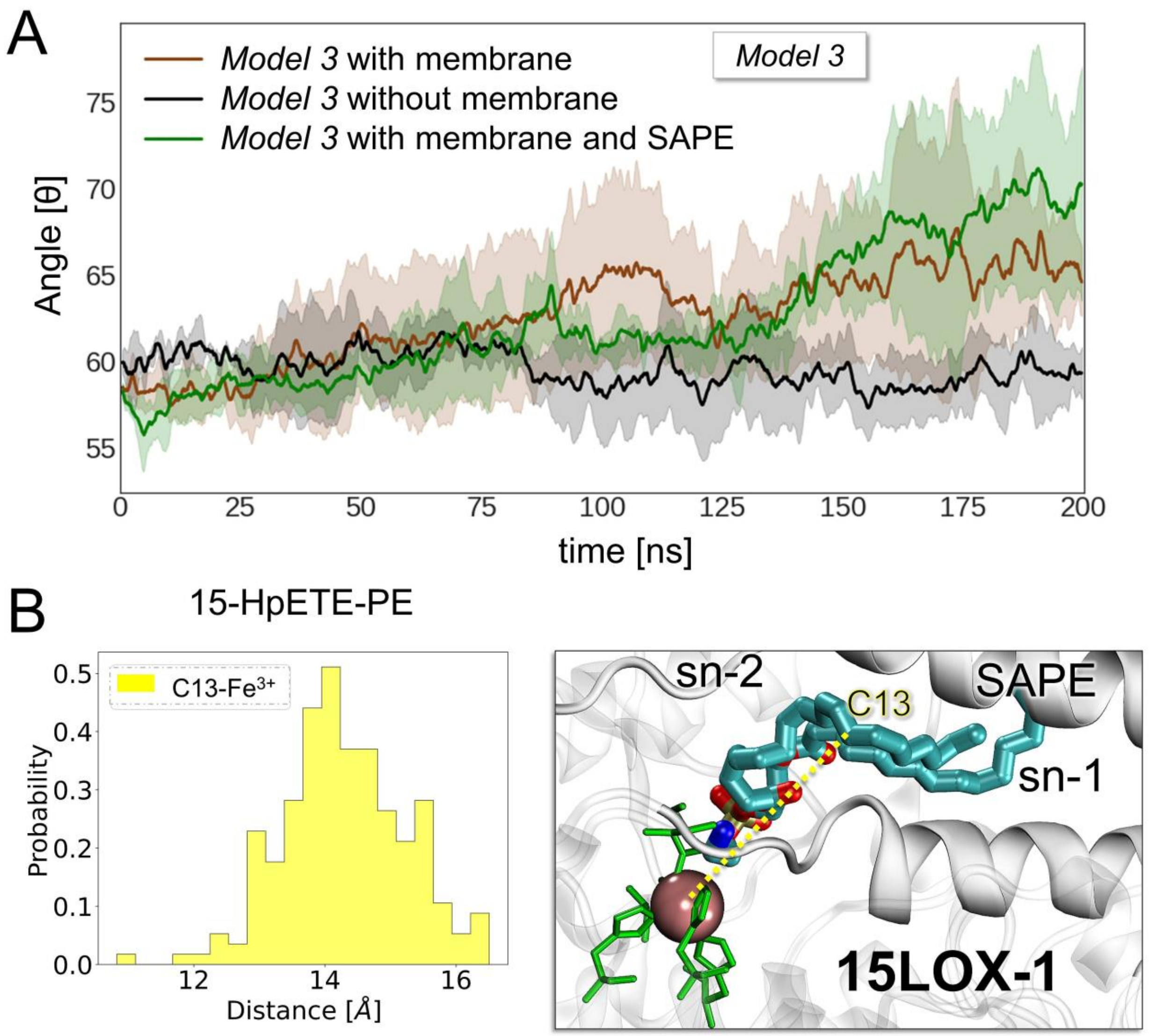

Supplementary Figure S8. (A) Time evolution of the angle θ in *Model 3* for the 15LOX-1/PEBP1 complex with different components. Average curves are shown in *black* (15LOX-1/PEBP1), in *brown* (15LOX-1/PEBP1+membrane) and in *green* (15LOX-1/PEBP1+membrane+SAPE). Corresponding shadows denote standard deviations computed from multiple independent MD runs. **(B)** Probability distribution of the distance between iron and C13 carbon of SAPE which contains potential hydrogen donors for the reaction catalyzed by 15LOX-1 for the run in which opening of the complex was the smallest. *On the right,* the close view of the pose of SAPE and the distance between C13 and the iron are shown (~14 Å). Iron is coordinated with three catalytic residues, H365, H540, H544 and I662 shown in *green sticks*.

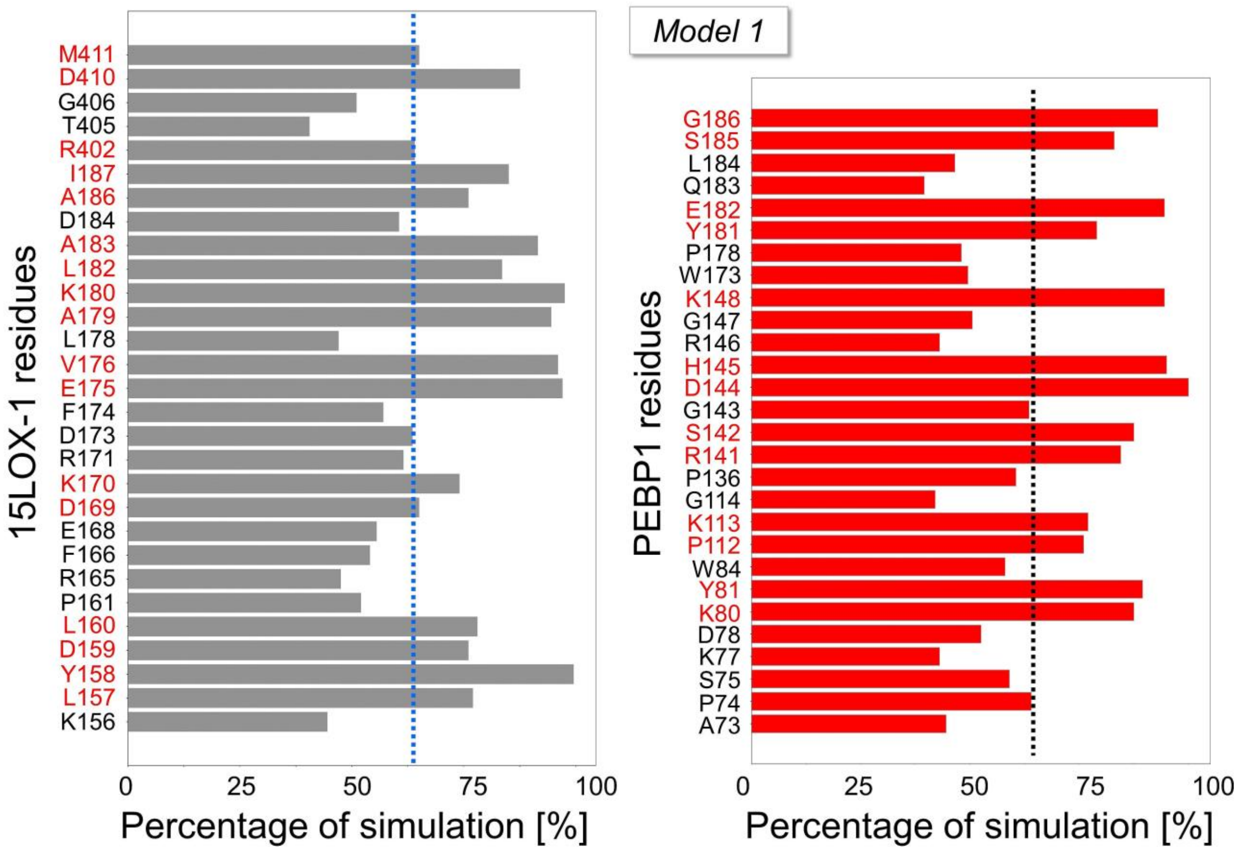

Supplementary Figure S9. PEBP1-15LOX-1 interactions in the presence of membrane. Histograms of counts for the most frequent interactions (< 5 Å) between PEBP1 and 15LOX-1 in *Model 1* during MD simulations with the membrane. Histograms are normalized to the percentage of simulation (100% = 250 ns multiplied by 3 runs where the opening was observed). The most frequent contacts (> 60% occurrence) for both proteins, 15LOX-1 and PEBP1, are labeled in *red*.

Supplementary Tables

Supplementary Table 1. List of 15-LOX-1 residues showing the highest propensity to engage in interfacial interactions with PEBP1.

Dark red	Y15, W40, R43, W76, F166, E168,R171,F174, L185, K188, D189, W197, F203, R205, F207, W208, Q211,Q416, W553, F583-Q585, W594, Q595, R598-Q600
Light red	L14, F77, W90, W99, R165, L167,D169, K170, V172, D173, E175, V176, L178, K180,L182, D184, I187, S190-T195, D202, I206, K213, E216, R219, L290, R402, I413, I417, L548, D549, P601

Supplementary Table 2. System setup for molecular dynamics simulations.

	**15LOX-1/PEBP1**	**15LOX-1/PEBP1 +membrane**	**15LOX-1 /PEBP1 + SAPE +membrane**	**15LOX-1/PEBP1^{mut} +SAPE +membrane**
Model 1	3 runs (200 ns each)	5 runs (250 ns each)	3 runs (200 ns each)	5 runs (75 ns each) for each of the mutants P112E, H86A, H86E, P74L
Model 2	3 runs (200 ns each)	5 runs (250 ns each)	-	-
Model 3	3 runs (200 ns each)	5 runs (250 ns each)	3 runs (200 ns each)	-
Model 4	2 runs (200 ns each)	-	-	-
