# Supplementary figures and images for: Membrane regulation of 15LOX-1/PEBP1 complex prompts the generation of ferroptotic signals, oxygenated PEs
Source: Free Radic Biol Med. Author manuscript; Available in PMC 2024 Nov 1. (PMC10952060; doi:10.1016/j.freeradbiomed.2023.09.001)

## Slide 1
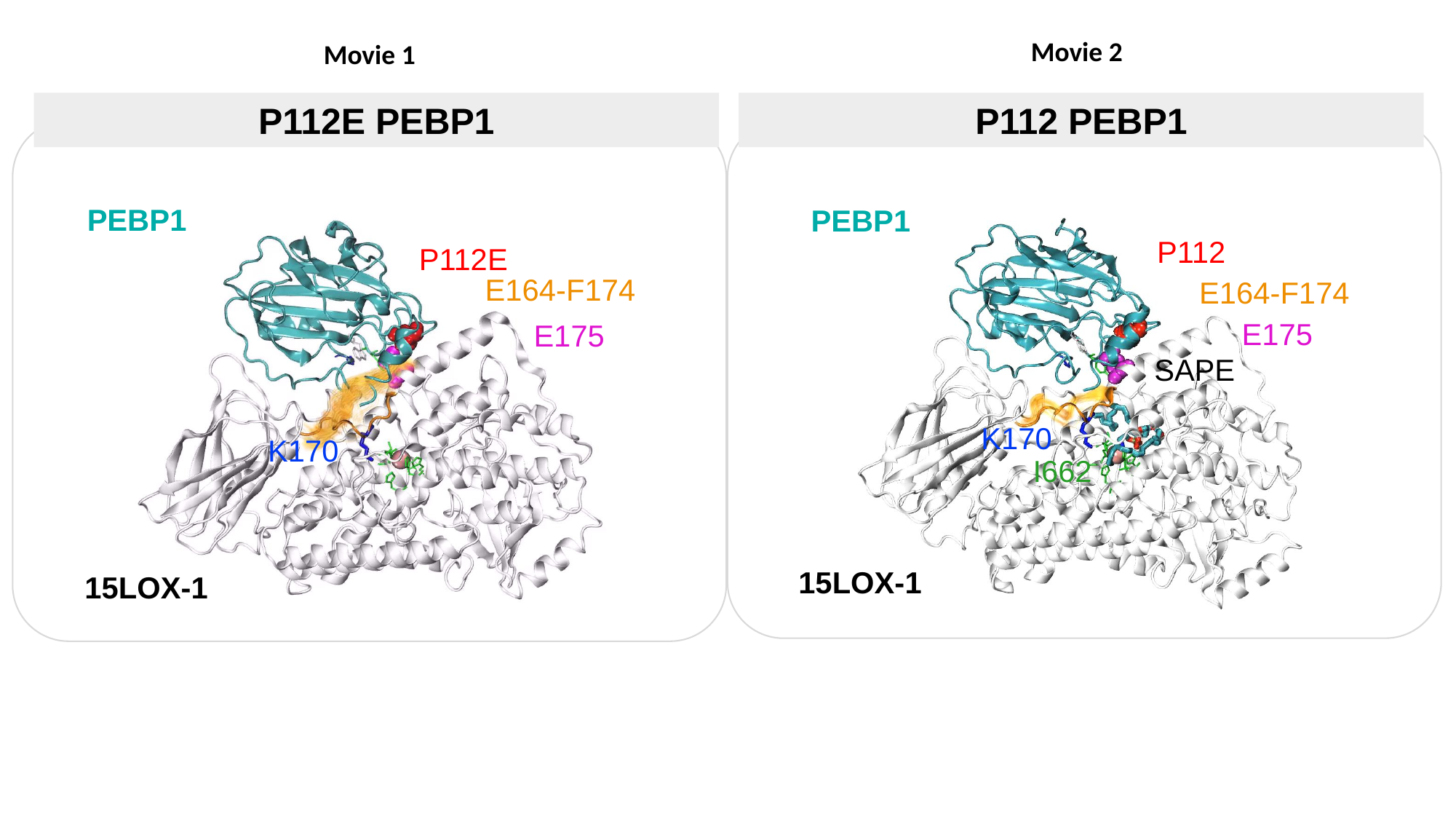

Movie 2
Movie 1
P112E PEBP1
P112 PEBP1
PEBP1
PEBP1
P112
P112E
E164-F174
E164-F174
E175
E175
SAPE
K170
K170
I662
15LOX-1
15LOX-1

Supplement: movies [file NIHMS1972082-supplement-movies.zip › FRB_16157_movies/4___Movies_15LOX1_PEBP1.pptx]
